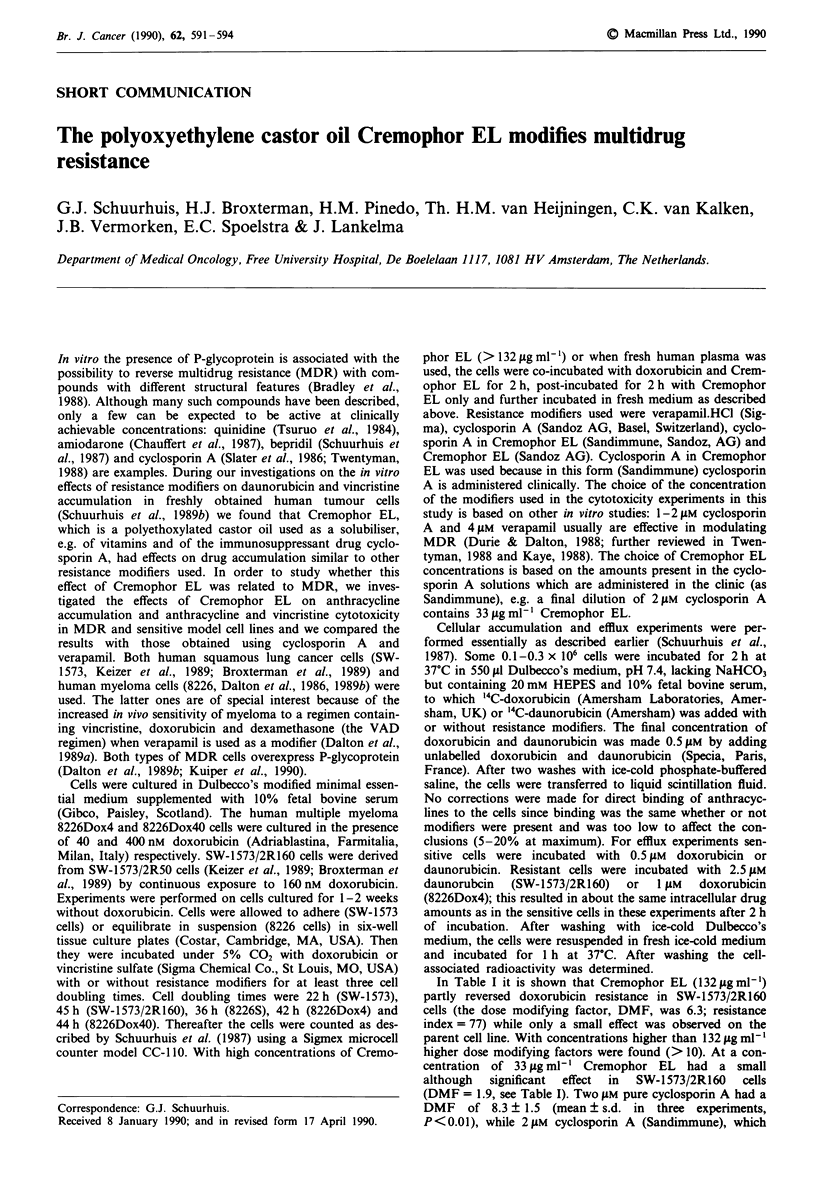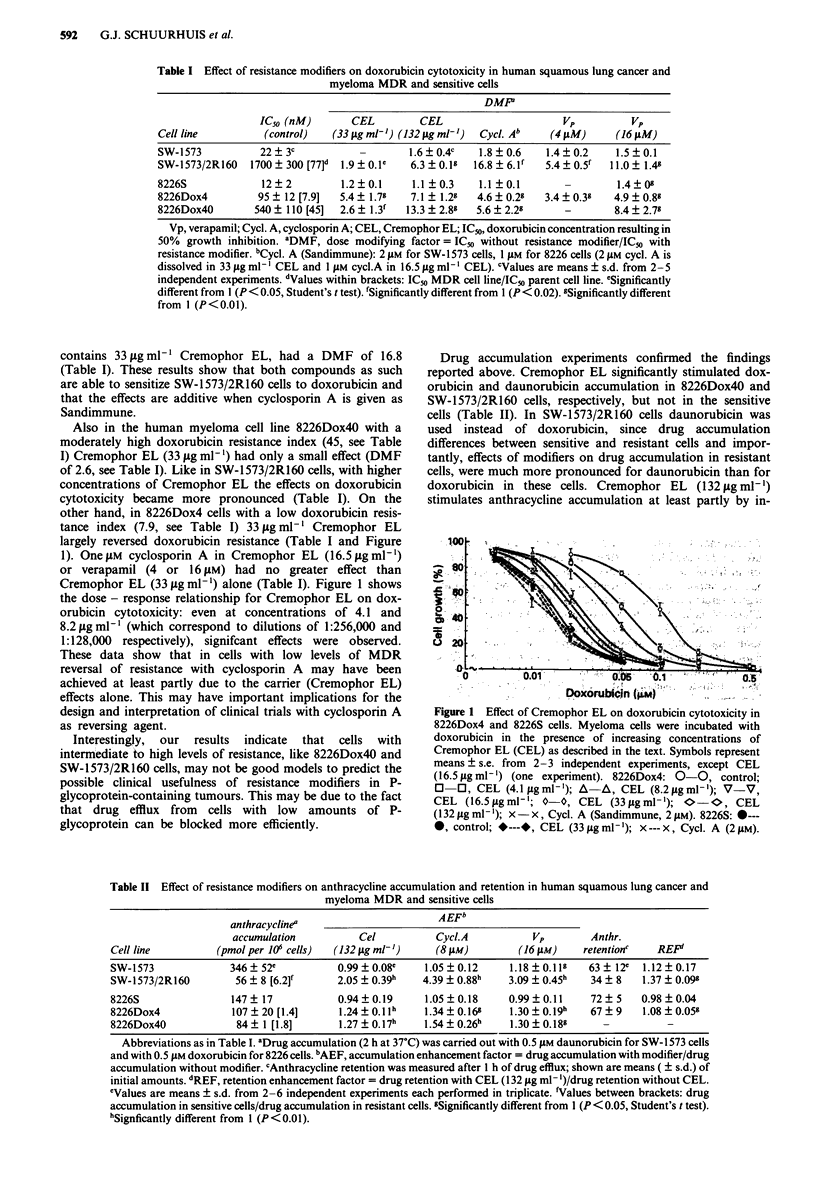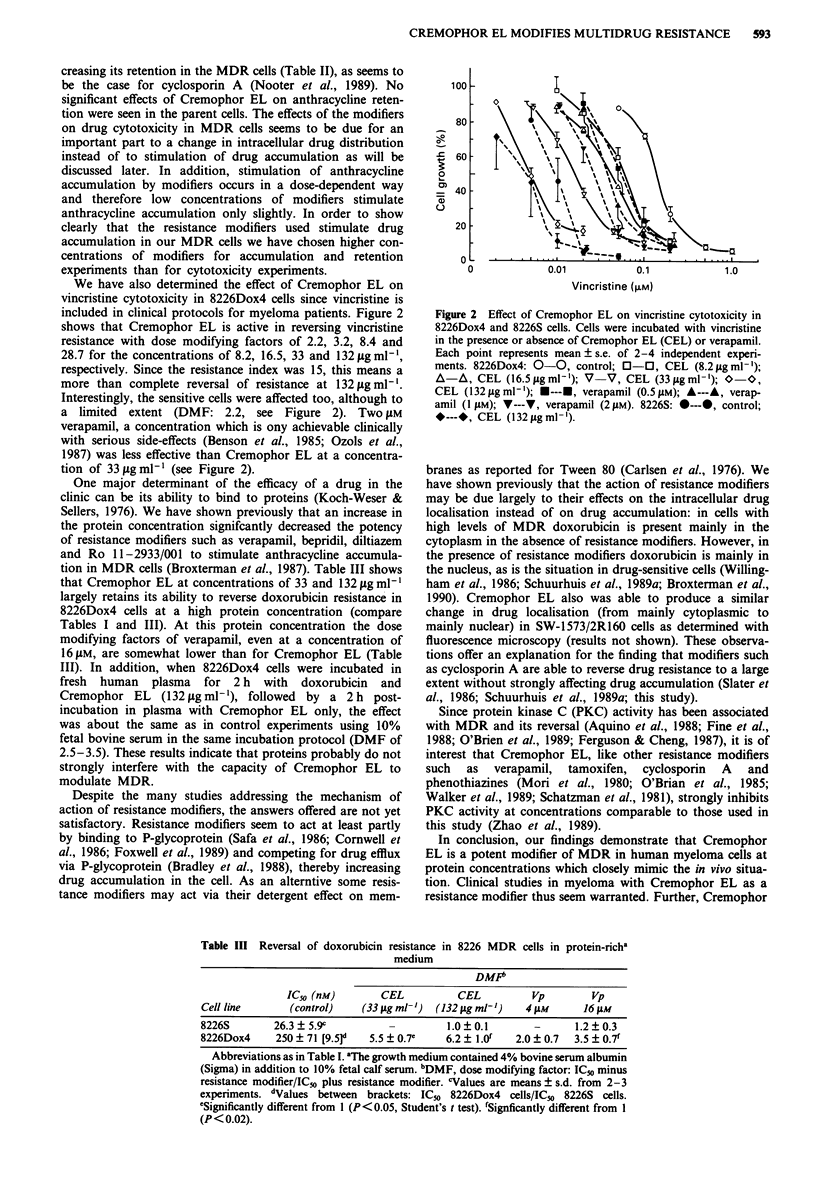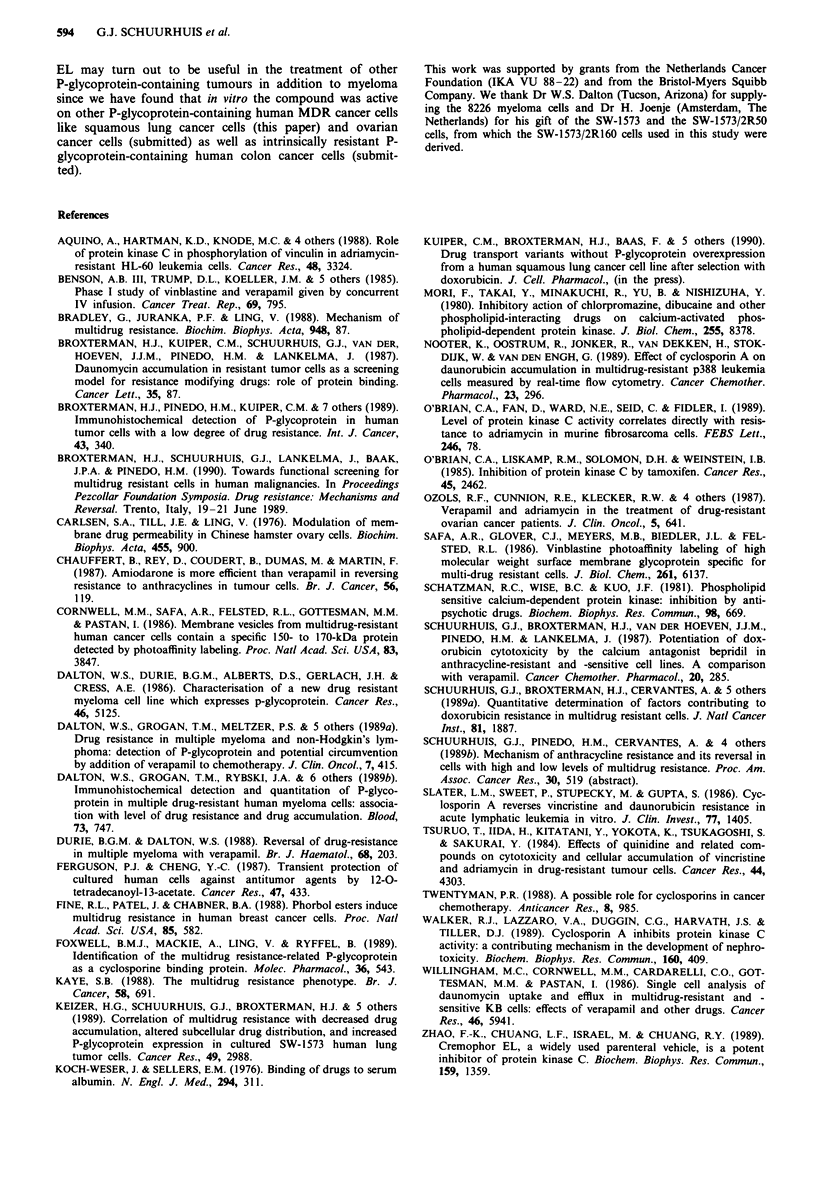# The polyoxyethylene castor oil Cremophor EL modifies multidrug resistance.

**DOI:** 10.1038/bjc.1990.335

**Published:** 1990-10

**Authors:** G. J. Schuurhuis, H. J. Broxterman, H. M. Pinedo, T. H. van Heijningen, C. K. van Kalken, J. B. Vermorken, E. C. Spoelstra, J. Lankelma

**Affiliations:** Department of Medical Oncology, Free University Hospital, Amsterdam, The Netherlands.


Br. J. Cancer (1990), 62, 591–594 © Macmillan Press Ltd., 1990

SHORT COMMUNICATION

### The polyoxyethylene castor oil Cremophor EL modifies multidrug resistance

G.J. Schuurhuis, H.J. Broxterman, H.M. Pinedo, Th. H.M. van Heijningen, C.K. van Kalken, J.B. Vermorken, E.C. Spoelstra & J. Lankelma

*Department of Medical Oncology, Free University Hospital, De Boelelaan 1117, 1081 HV Amsterdam, The Netherlands.*

*In vitro* the presence of P-glycoprotein is associated with the possibility to reverse multidrug resistance (MDR) with compounds with different structural features (Bradley *et al.*, 1988). Although many such compounds have been described, only a few can be expected to be active at clinically achievable concentrations: quinidine (Tsuruo *et al.*, 1984), amiodarone (Chauffert *et al.*, 1987), bepridil (Schuurhuis *et al.*, 1987) and cyclosporin A (Slater *et al.*, 1986; Twentyman, 1988) are examples. During our investigations on the *in vitro* effects of resistance modifiers on daunorubicin and vincristine accumulation in freshly obtained human tumour cells (Schuurhuis *et al.*, 1989*b*) we found that Cremophor EL, which is a polyethoxylated castor oil used as a solubiliser, e.g. of vitamins and of the immunosuppressant drug cyclosporin A, had effects on drug accumulation similar to other resistance modifiers used. In order to study whether this effect of Cremophor EL was related to MDR, we investigated the effects of Cremophor EL on anthracycline accumulation and anthracycline and vincristine cytotoxicity in MDR and sensitive model cell lines and we compared the results with those obtained using cyclosporin A and verapamil. Both human squamous lung cancer cells (SW-1573, Keizer *et al.*, 1989; Broxterman *et al.*, 1989) and human myeloma cells (8226, Dalton *et al.*, 1986, 1989*b*) were used. The latter ones are of special interest because of the increased *in vivo* sensitivity of myeloma to a regimen containing vincristine, doxorubicin and dexamethasone (the VAD regimen) when verapamil is used as a modifier (Dalton *et al.*, 1989*a*). Both types of MDR cells overexpress P-glycoprotein (Dalton *et al.*, 1989*b*; Kuiper *et al.*, 1990).

Cells were cultured in Dulbecco's modified minimal essential medium supplemented with 10% fetal bovine serum (Gibco, Paisley, Scotland). The human multiple myeloma 8226Dox4 and 8226Dox40 cells were cultured in the presence of 40 and 400 nM doxorubicin (Adriablastina, Farmitalia, Milan, Italy) respectively. SW-1573/2R160 cells were derived from SW-1573/2R50 cells (Keizer *et al.*, 1989; Broxterman *et al.*, 1989) by continuous exposure to 160 nM doxorubicin. Experiments were performed on cells cultured for 1–2 weeks without doxorubicin. Cells were allowed to adhere (SW-1573 cells) or equilibrate in suspension (8226 cells) in six-well tissue culture plates (Costar, Cambridge, MA, USA). Then they were incubated under 5% $CO_2$ with doxorubicin or vincristine sulfate (Sigma Chemical Co., St Louis, MO, USA) with or without resistance modifiers for at least three cell doubling times. Cell doubling times were 22 h (SW-1573), 45 h (SW-1573/2R160), 36 h (8226S), 42 h (8226Dox4) and 44 h (8226Dox40). Thereafter the cells were counted as described by Schuurhuis *et al.* (1987) using a Sigmex microcell counter model CC-110. With high concentrations of Cremophor EL (> 132 μg ml$^{-1}$) or when fresh human plasma was used, the cells were co-incubated with doxorubicin and Cremophor EL for 2 h, post-incubated for 2 h with Cremophor EL only and further incubated in fresh medium as described above. Resistance modifiers used were verapamil.HCl (Sigma), cyclosporin A (Sandoz AG, Basel, Switzerland), cyclosporin A in Cremophor EL (Sandimmune, Sandoz, AG) and Cremophor EL (Sandoz AG). Cyclosporin A in Cremophor EL was used because in this form (Sandimmune) cyclosporin A is administered clinically. The choice of the concentration of the modifiers used in the cytotoxicity experiments in this study is based on other *in vitro* studies: 1–2 μM cyclosporin A and 4 μM verapamil usually are effective in modulating MDR (Durie & Dalton, 1988; further reviewed in Twentyman, 1988 and Kaye, 1988). The choice of Cremophor EL concentrations is based on the amounts present in the cyclosporin A solutions which are administered in the clinic (as Sandimmune), e.g. a final dilution of 2 μM cyclosporin A contains 33 μg ml$^{-1}$ Cremophor EL.

Cellular accumulation and efflux experiments were performed essentially as described earlier (Schuurhuis *et al.*, 1987). Some 0.1–0.3 × 10$^6$ cells were incubated for 2 h at 37°C in 550 μl Dulbecco's medium, pH 7.4, lacking $NaHCO_3$ but containing 20 mM HEPES and 10% fetal bovine serum, to which $^{14}$C-doxorubicin (Amersham Laboratories, Amersham, UK) or $^{14}$C-daunorubicin (Amersham) was added with or without resistance modifiers. The final concentration of doxorubicin and daunorubicin was made 0.5 μM by adding unlabelled doxorubicin and daunorubicin (Specia, Paris, France). After two washes with ice-cold phosphate-buffered saline, the cells were transferred to liquid scintillation fluid. No corrections were made for direct binding of anthracyclines to the cells since binding was the same whether or not modifiers were present and was too low to affect the conclusions (5–20% at maximum). For efflux experiments sensitive cells were incubated with 0.5 μM doxorubicin or daunorubicin. Resistant cells were incubated with 2.5 μM daunorubcin (SW-1573/2R160) or 1 μM doxorubicin (8226Dox4); this resulted in about the same intracellular drug amounts as in the sensitive cells in these experiments after 2 h of incubation. After washing with ice-cold Dulbecco's medium, the cells were resuspended in fresh ice-cold medium and incubated for 1 h at 37°C. After washing the cell-associated radioactivity was determined.

In Table I it is shown that Cremophor EL (132 μg ml$^{-1}$) partly reversed doxorubicin resistance in SW-1573/2R160 cells (the dose modifying factor, DMF, was 6.3; resistance index = 77) while only a small effect was observed on the parent cell line. With concentrations higher than 132 μg ml$^{-1}$ higher dose modifying factors were found (> 10). At a concentration of 33 μg ml$^{-1}$ Cremophor EL had a small although significant effect in SW-1573/2R160 cells (DMF = 1.9, see Table I). Two μM pure cyclosporin A had a DMF of 8.3 ± 1.5 (mean ± s.d. in three experiments, $P < 0.01$), while 2 μM cyclosporin A (Sandimmune), which

Correspondence: G.J. Schuurhuis.
Received 8 January 1990; and in revised form 17 April 1990.

**Table I** Effect of resistance modifiers on doxorubicin cytotoxicity in human squamous lung cancer and myeloma MDR and sensitive cells

| Cell line | $IC_{50}$ (nM) (control) | DMF[a] | | | | |
|---|---|---|---|---|---|---|
| | | CEL (33 μg ml$^{-1}$) | CEL (132 μg ml$^{-1}$) | Cycl. A[b] | $V_P$ (4 μM) | $V_P$ (16 μM) |
| SW-1573 | 22 ± 3[c] | – | 1.6 ± 0.4[c] | 1.8 ± 0.6 | 1.4 ± 0.2 | 1.5 ± 0.1 |
| SW-1573/2R160 | 1700 ± 300 [77][d] | 1.9 ± 0.1[e] | 6.3 ± 0.1[g] | 16.8 ± 6.1[f] | 5.4 ± 0.5[f] | 11.0 ± 1.4[g] |
| 8226S | 12 ± 2 | 1.2 ± 0.1 | 1.1 ± 0.3 | 1.1 ± 0.1 | – | 1.4 ± 0[g] |
| 8226Dox4 | 95 ± 12 [7.9] | 5.4 ± 1.7[g] | 7.1 ± 1.2[g] | 4.6 ± 0.2[g] | 3.4 ± 0.3[g] | 4.9 ± 0.8[g] |
| 8226Dox40 | 540 ± 110 [45] | 2.6 ± 1.3[f] | 13.3 ± 2.8[g] | 5.6 ± 2.2[g] | – | 8.4 ± 2.7[g] |

Vp, verapamil; Cycl. A, cyclosporin A; CEL, Cremophor EL; $IC_{50}$, doxorubicin concentration resulting in 50% growth inhibition. [a]DMF, dose modifying factor = $IC_{50}$ without resistance modifier/$IC_{50}$ with resistance modifier. [b]Cycl. A (Sandimmune): 2 μM for SW-1573 cells, 1 μM for 8226 cells (2 μM cycl. A is dissolved in 33 μg ml$^{-1}$ CEL and 1 μM cycl.A in 16.5 μg ml$^{-1}$ CEL). [c]Values are means ± s.d. from 2–5 independent experiments. [d]Values within brackets: $IC_{50}$ MDR cell line/$IC_{50}$ parent cell line. [e]Significantly different from 1 ($P < 0.05$, Student's *t* test). [f]Significantly different from 1 ($P < 0.02$). [g]Significantly different from 1 ($P < 0.01$).

contains 33 μg ml$^{-1}$ Cremophor EL, had a DMF of 16.8 (Table I). These results show that both compounds as such are able to sensitize SW-1573/2R160 cells to doxorubicin and that the effects are additive when cyclosporin A is given as Sandimmune.

Also in the human myeloma cell line 8226Dox40 with a moderately high doxorubicin resistance index (45, see Table I) Cremophor EL (33 μg ml$^{-1}$) had only a small effect (DMF of 2.6, see Table I). Like in SW-1573/2R160 cells, with higher concentrations of Cremophor EL the effects on doxorubicin cytotoxicity became more pronounced (Table I). On the other hand, in 8226Dox4 cells with a low doxorubicin resistance index (7.9, see Table I) 33 μg ml$^{-1}$ Cremophor EL largely reversed doxorubicin resistance (Table I and Figure 1). One μM cyclosporin A in Cremophor EL (16.5 μg ml$^{-1}$) or verapamil (4 or 16 μM) had no greater effect than Cremophor EL (33 μg ml$^{-1}$) alone (Table I). Figure 1 shows the dose – response relationship for Cremophor EL on doxorubicin cytotoxicity: even at concentrations of 4.1 and 8.2 μg ml$^{-1}$ (which correspond to dilutions of 1:256,000 and 1:128,000 respectively), signifcant effects were observed. These data show that in cells with low levels of MDR reversal of resistance with cyclosporin A may have been achieved at least partly due to the carrier (Cremophor EL) effects alone. This may have important implications for the design and interpretation of clinical trials with cyclosporin A as reversing agent.

Interestingly, our results indicate that cells with intermediate to high levels of resistance, like 8226Dox40 and SW-1573/2R160 cells, may not be good models to predict the possible clinical usefulness of resistance modifiers in P-glycoprotein-containing tumours. This may be due to the fact that drug efflux from cells with low amounts of P-glycoprotein can be blocked more efficiently.

Drug accumulation experiments confirmed the findings reported above. Cremophor EL significantly stimulated doxorubicin and daunorubicin accumulation in 8226Dox40 and SW-1573/2R160 cells, respectively, but not in the sensitive cells (Table II). In SW-1573/2R160 cells daunorubicin was used instead of doxorubicin, since drug accumulation differences between sensitive and resistant cells and importantly, effects of modifiers on drug accumulation in resistant cells, were much more pronounced for daunorubicin than for doxorubicin in these cells. Cremophor EL (132 μg ml$^{-1}$) stimulates anthracycline accumulation at least partly by in-

**Figure 1** Effect of Cremophor EL on doxorubicin cytotoxicity in 8226Dox4 and 8226S cells. Myeloma cells were incubated with doxorubicin in the presence of increasing concentrations of Cremophor EL (CEL) as described in the text. Symbols represent means ± s.e. from 2–3 independent experiments, except CEL (16.5 μg ml$^{-1}$) (one experiment). 8226Dox4: O—O, control; □—□, CEL (4.1 μg ml$^{-1}$); Δ—Δ, CEL (8.2 μg ml$^{-1}$); ∇—∇, CEL (16.5 μg ml$^{-1}$; ◇—◇, CEL (33 μg ml$^{-1}$); ◇—◇, CEL (132 μg ml$^{-1}$); ×—×, Cycl. A (Sandimmune, 2 μM). 8226S: ●---●, control; ◆---◆, CEL (33 μg ml$^{-1}$); ×---×, Cycl. A (2 μM).

**Table II** Effect of resistance modifiers on anthracycline accumulation and retention in human squamous lung cancer and myeloma MDR and sensitive cells

| Cell line | anthracycline[a] accumulation (pmol per $10^6$ cells) | AEF[b] | | | Anthr. retention[c] | REF[d] |
|---|---|---|---|---|---|---|
| | | Cel (132 μg ml$^{-1}$) | Cycl.A (8 μM) | $V_P$ (16 μM) | | |
| SW-1573 | 346 ± 52[e] | 0.99 ± 0.08[e] | 1.05 ± 0.12 | 1.18 ± 0.11[g] | 63 ± 12[e] | 1.12 ± 0.17 |
| SW-1573/2R160 | 56 ± 8 [6.2][f] | 2.05 ± 0.39[h] | 4.39 ± 0.88[h] | 3.09 ± 0.45[h] | 34 ± 8 | 1.37 ± 0.09[g] |
| 8226S | 147 ± 17 | 0.94 ± 0.19 | 1.05 ± 0.18 | 0.99 ± 0.11 | 72 ± 5 | 0.98 ± 0.04 |
| 8226Dox4 | 107 ± 20 [1.4] | 1.24 ± 0.11[h] | 1.34 ± 0.16[g] | 1.30 ± 0.19[h] | 67 ± 9 | 1.08 ± 0.05[g] |
| 8226Dox40 | 84 ± 1 [1.8] | 1.27 ± 0.17[h] | 1.54 ± 0.26[h] | 1.30 ± 0.18[g] | – | – |

Abbreviations as in Table I. [a]Drug accumulation (2 h at 37°C) was carried out with 0.5 μM daunorubicin for SW-1573 cells and with 0.5 μM doxorubicin for 8226 cells. [b]AEF, accumulation enhancement factor = drug accumulation with modifier/drug accumulation without modifier. [c]Anthracycline retention was measured after 1 h of drug efflux; shown are means ( ± s.d.) of initial amounts. [d]REF, retention enhancement factor = drug retention with CEL (132 μg ml$^{-1}$)/drug retention without CEL. [e]Values are means ± s.d. from 2–6 independent experiments each performed in triplicate. [f]Values between brackets: drug accumulation in sensitive cells/drug accumulation in resistant cells. [g]Significantly different from 1 ($P < 0.05$, Student's *t* test). [h]Signficantly different from 1 ($P < 0.01$).

creasing its retention in the MDR cells (Table II), as seems to be the case for cyclosporin A (Nooter *et al.*, 1989). No significant effects of Cremophor EL on anthracycline retention were seen in the parent cells. The effects of the modifiers on drug cytotoxicity in MDR cells seems to be due for an important part to a change in intracellular drug distribution instead of to stimulation of drug accumulation as will be discussed later. In addition, stimulation of anthracycline accumulation by modifiers occurs in a dose-dependent way and therefore low concentrations of modifiers stimulate anthracycline accumulation only slightly. In order to show clearly that the resistance modifiers used stimulate drug accumulation in our MDR cells we have chosen higher concentrations of modifiers for accumulation and retention experiments than for cytotoxicity experiments.

We have also determined the effect of Cremophor EL on vincristine cytotoxicity in 8226Dox4 cells since vincristine is included in clinical protocols for myeloma patients. Figure 2 shows that Cremophor EL is active in reversing vincristine resistance with dose modifying factors of 2.2, 3.2, 8.4 and 28.7 for the concentrations of 8.2, 16.5, 33 and 132 $\mu g\ ml^{-1}$, respectively. Since the resistance index was 15, this means a more than complete reversal of resistance at 132 $\mu g\ ml^{-1}$. Interestingly, the sensitive cells were affected too, although to a limited extent (DMF: 2.2, see Figure 2). Two μM verapamil, a concentration which is ony achievable clinically with serious side-effects (Benson *et al.*, 1985; Ozols *et al.*, 1987) was less effective than Cremophor EL at a concentration of 33 $\mu g\ ml^{-1}$ (see Figure 2).

One major determinant of the efficacy of a drug in the clinic can be its ability to bind to proteins (Koch-Weser & Sellers, 1976). We have shown previously that an increase in the protein concentration signifcantly decreased the potency of resistance modifiers such as verapamil, bepridil, diltiazem and Ro 11-2933/001 to stimulate anthracycline accumulation in MDR cells (Broxterman *et al.*, 1987). Table III shows that Cremophor EL at concentrations of 33 and 132 $\mu g\ ml^{-1}$ largely retains its ability to reverse doxorubicin resistance in 8226Dox4 cells at a high protein concentration (compare Tables I and III). At this protein concentration the dose modifying factors of verapamil, even at a concentration of 16 μM, are somewhat lower than for Cremophor EL (Table III). In addition, when 8226Dox4 cells were incubated in fresh human plasma for 2 h with doxorubicin and Cremophor EL (132 $\mu g\ ml^{-1}$), followed by a 2 h post-incubation in plasma with Cremophor EL only, the effect was about the same as in control experiments using 10% fetal bovine serum in the same incubation protocol (DMF of 2.5–3.5). These results indicate that proteins probably do not strongly interfere with the capacity of Cremophor EL to modulate MDR.

Despite the many studies addressing the mechanism of action of resistance modifiers, the answers offered are not yet satisfactory. Resistance modifiers seem to act at least partly by binding to P-glycoprotein (Safa *et al.*, 1986; Cornwell *et al.*, 1986; Foxwell *et al.*, 1989) and competing for drug efflux via P-glycoprotein (Bradley *et al.*, 1988), thereby increasing drug accumulation in the cell. As an alterntive some resistance modifiers may act via their detergent effect on membranes as reported for Tween 80 (Carlsen *et al.*, 1976). We have shown previously that the action of resistance modifiers may be due largely to their effects on the intracellular drug localisation instead of on drug accumulation: in cells with high levels of MDR doxorubicin is present mainly in the cytoplasm in the absence of resistance modifiers. However, in the presence of resistance modifiers doxorubicin is mainly in the nucleus, as is the situation in drug-sensitive cells (Willingham *et al.*, 1986; Schuurhuis *et al.*, 1989*a*; Broxterman *et al.*, 1990). Cremophor EL also was able to produce a similar change in drug localisation (from mainly cytoplasmic to mainly nuclear) in SW-1573/2R160 cells as determined with fluorescence microscopy (results not shown). These observations offer an explanation for the finding that modifiers such as cyclosporin A are able to reverse drug resistance to a large extent without strongly affecting drug accumulation (Slater *et al.*, 1986; Schuurhuis *et al.*, 1989*a*; this study).

**Figure 2** Effect of Cremophor EL on vincristine cytotoxicity in 8226Dox4 and 8226S cells. Cells were incubated with vincristine in the presence or absence of Cremophor EL (CEL) or verapamil. Each point represents mean ± s.e. of 2–4 independent experiments. 8226Dox4: O—O, control; □—□, CEL (8.2 $\mu g\ ml^{-1}$); Δ—Δ, CEL (16.5 $\mu g\ ml^{-1}$); ∇—∇, CEL (33 $\mu g\ ml^{-1}$); ◇—◇, CEL (132 $\mu g\ ml^{-1}$); ■---■, verapamil (0.5 μM); ▲---▲, verapamil (1 μM); ▼---▼, verapamil (2 μM). 8226S: ●---●, control; ◆---◆, CEL (132 $\mu g\ ml^{-1}$).

Since protein kinase C (PKC) activity has been associated with MDR and its reversal (Aquino *et al.*, 1988; Fine *et al.*, 1988; O'Brien *et al.*, 1989; Ferguson & Cheng, 1987), it is of interest that Cremophor EL, like other resistance modifiers such as verapamil, tamoxifen, cyclosporin A and phenothiazines (Mori *et al.*, 1980; O'Brian *et al.*, 1985; Walker *et al.*, 1989; Schatzman *et al.*, 1981), strongly inhibits PKC activity at concentrations comparable to those used in this study (Zhao *et al.*, 1989).

In conclusion, our findings demonstrate that Cremophor EL is a potent modifier of MDR in human myeloma cells at protein concentrations which closely mimic the *in vivo* situation. Clinical studies in myeloma with Cremophor EL as a resistance modifier thus seem warranted. Further, Cremophor

**Table III** Reversal of doxorubicin resistance in 8226 MDR cells in protein-rich[a] medium

| | | DMF[b] | | | |
|---|---|---|---|---|---|
| *Cell line* | *$IC_{50}$ (nM) (control)* | *CEL (33 $\mu g\ ml^{-1}$)* | *CEL (132 $\mu g\ ml^{-1}$)* | *Vp 4 μM* | *Vp 16 μM* |
| 8226S | 26.3 ± 5.9[c] | – | 1.0 ± 0.1 | – | 1.2 ± 0.3 |
| 8226Dox4 | 250 ± 71 [9.5][d] | 5.5 ± 0.7[e] | 6.2 ± 1.0[f] | 2.0 ± 0.7 | 3.5 ± 0.7[f] |

Abbreviations as in Table I. [a]The growth medium contained 4% bovine serum albumin (Sigma) in addition to 10% fetal calf serum. [b]DMF, dose modifying factor: $IC_{50}$ minus resistance modifier/$IC_{50}$ plus resistance modifier. [c]Values are means ± s.d. from 2–3 experiments. [d]Values between brackets: $IC_{50}$ 8226Dox4 cells/$IC_{50}$ 8226S cells. [e]Significantly different from 1 ($P<0.05$, Student's *t* test). [f]Signficantly different from 1 ($P<0.02$).

EL may turn out to be useful in the treatment of other P-glycoprotein-containing tumours in addition to myeloma since we have found that *in vitro* the compound was active on other P-glycoprotein-containing human MDR cancer cells like squamous lung cancer cells (this paper) and ovarian cancer cells (submitted) as well as intrinsically resistant P-glycoprotein-containing human colon cancer cells (submitted).

This work was supported by grants from the Netherlands Cancer Foundation (IKA VU 88-22) and from the Bristol-Myers Squibb Company. We thank Dr W.S. Dalton (Tucson, Arizona) for supplying the 8226 myeloma cells and Dr H. Joenje (Amsterdam, The Netherlands) for his gift of the SW-1573 and the SW-1573/2R50 cells, from which the SW-1573/2R160 cells used in this study were derived.

### References

AQUINO, A., HARTMAN, K.D., KNODE, M.C. & 4 others (1988). Role of protein kinase C in phosphorylation of vinculin in adriamycin-resistant HL-60 leukemia cells. *Cancer Res.*, **48**, 3324.

BENSON, A.B. III, TRUMP, D.L., KOELLER, J.M. & 5 others (1985). Phase I study of vinblastine and verapamil given by concurrent IV infusion. *Cancer Treat. Rep.*, **69**, 795.

BRADLEY, G., JURANKA, P.F. & LING, V. (1988). Mechanism of multidrug resistance. *Biochim. Biophys. Acta*, **948**, 87.

BROXTERMAN, H.J., KUIPER, C.M., SCHUURHUIS, G.J., VAN DER HOEVEN, J.J.M., PINEDO, H.M. & LANKELMA, J. (1987). Daunomycin accumulation in resistant tumor cells as a screening model for resistance modifying drugs: role of protein binding. *Cancer Lett.*, **35**, 87.

BROXTERMAN, H.J., PINEDO, H.M., KUIPER, C.M. & 7 others (1989). Immunohistochemical detection of P-glycoprotein in human tumor cells with a low degree of drug resistance. *Int. J. Cancer*, **43**, 340.

BROXTERMAN, H.J., SCHUURHUIS, G.J., LANKELMA, J., BAAK, J.P.A. & PINEDO, H.M. (1990). Towards functional screening for multidrug resistant cells in human malignancies. In *Proceedings Pezcollar Foundation Symposia. Drug resistance: Mechanisms and Reversal*. Trento, Italy, 19-21 June 1989.

CARLSEN, S.A., TILL, J.E. & LING, V. (1976). Modulation of membrane drug permeability in Chinese hamster ovary cells. *Biochim. Biophys. Acta*, **455**, 900.

CHAUFFERT, B., REY, D., COUDERT, B., DUMAS, M. & MARTIN, F. (1987). Amiodarone is more efficient than verapamil in reversing resistance to anthracyclines in tumour cells. *Br. J. Cancer*, **56**, 119.

CORNWELL, M.M., SAFA, A.R., FELSTED, R.L., GOTTESMAN, M.M. & PASTAN, I. (1986). Membrane vesicles from multidrug-resistant human cancer cells contain a specific 150- to 170-kDa protein detected by photoaffinity labeling. *Proc. Natl Acad. Sci. USA*, **83**, 3847.

DALTON, W.S., DURIE, B.G.M., ALBERTS, D.S., GERLACH, J.H. & CRESS, A.E. (1986). Characterisation of a new drug resistant myeloma cell line which expresses p-glycoprotein. *Cancer Res.*, **46**, 5125.

DALTON, W.S., GROGAN, T.M., MELTZER, P.S. & 5 others (1989*a*). Drug resistance in multiple myeloma and non-Hodgkin's lymphoma: detection of P-glycoprotein and potential circumvention by addition of verapamil to chemotherapy. *J. Clin. Oncol.*, **7**, 415.

DALTON, W.S., GROGAN, T.M., RYBSKI, J.A. & 6 others (1989*b*). Immunohistochemical detection and quantitation of P-glycoprotein in multiple drug-resistant human myeloma cells: association with level of drug resistance and drug accumulation. *Blood*, **73**, 747.

DURIE, B.G.M. & DALTON, W.S. (1988). Reversal of drug-resistance in multiple myeloma with verapamil. *Br. J. Haematol.*, **68**, 203.

FERGUSON, P.J. & CHENG, Y.-C. (1987). Transient protection of cultured human cells against antitumor agents by 12-O-tetradecanoyl-13-acetate. *Cancer Res.*, **47**, 433.

FINE, R.L., PATEL, J. & CHABNER, B.A. (1988). Phorbol esters induce multidrug resistance in human breast cancer cells. *Proc. Natl Acad. Sci. USA*, **85**, 582.

FOXWELL, B.M.J., MACKIE, A., LING, V. & RYFFEL, B. (1989). Identification of the multidrug resistance-related P-glycoprotein as a cyclosporine binding protein. *Molec. Pharmacol.*, **36**, 543.

KAYE, S.B. (1988). The multidrug resistance phenotype. *Br. J. Cancer*, **58**, 691.

KEIZER, H.G., SCHUURHUIS, G.J., BROXTERMAN, H.J. & 5 others (1989). Correlation of multidrug resistance with decreased drug accumulation, altered subcellular drug distribution, and increased P-glycoprotein expression in cultured SW-1573 human lung tumor cells. *Cancer Res.*, **49**, 2988.

KOCH-WESER, J. & SELLERS, E.M. (1976). Binding of drugs to serum albumin. *N. Engl. J. Med.*, **294**, 311.

KUIPER, C.M., BROXTERMAN, H.J., BAAS, F. & 5 others (1990). Drug transport variants without P-glycoprotein overexpression from a human squamous lung cancer cell line after selection with doxorubicin. *J. Cell. Pharmacol.*, (in the press).

MORI, F., TAKAI, Y., MINAKUCHI, R., YU, B. & NISHIZUHA, Y. (1980). Inhibitory action of chlorpromazine, dibucaine and other phospholipid-interacting drugs on calcium-activated phospholipid-dependent protein kinase. *J. Biol. Chem.*, **255**, 8378.

NOOTER, K., OOSTRUM, R., JONKER, R., VAN DEKKEN, H., STOKDIJK, W. & VAN DEN ENGH, G. (1989). Effect of cyclosporin A on daunorubicin accumulation in multidrug-resistant p388 leukemia cells measured by real-time flow cytometry. *Cancer Chemother. Pharmacol.*, **23**, 296.

O'BRIAN, C.A., FAN, D., WARD, N.E., SEID, C. & FIDLER, I. (1989). Level of protein kinase C activity correlates directly with resistance to adriamycin in murine fibrosarcoma cells. *FEBS Lett.*, **246**, 78.

O'BRIAN, C.A., LISKAMP, R.M., SOLOMON, D.H. & WEINSTEIN, I.B. (1985). Inhibition of protein kinase C by tamoxifen. *Cancer Res.*, **45**, 2462.

OZOLS, R.F., CUNNION, R.E., KLECKER, R.W. & 4 others (1987). Verapamil and adriamycin in the treatment of drug-resistant ovarian cancer patients. *J. Clin. Oncol.*, **5**, 641.

SAFA, A.R., GLOVER, C.J., MEYERS, M.B., BIEDLER, J.L. & FELSTED, R.L. (1986). Vinblastine photoaffinity labeling of high molecular weight surface membrane glycoprotein specific for multi-drug resistant cells. *J. Biol. Chem.*, **261**, 6137.

SCHATZMAN, R.C., WISE, B.C. & KUO, J.F. (1981). Phospholipid sensitive calcium-dependent protein kinase: inhibition by antipsychotic drugs. *Biochem. Biophys. Res. Commun.*, **98**, 669.

SCHUURHUIS, G.J., BROXTERMAN, H.J., VAN DER HOEVEN, J.J.M., PINEDO, H.M. & LANKELMA, J. (1987). Potentiation of doxorubicin cytotoxicity by the calcium antagonist bepridil in anthracycline-resistant and -sensitive cell lines. A comparison with verapamil. *Cancer Chemother. Pharmacol.*, **20**, 285.

SCHUURHUIS, G.J., BROXTERMAN, H.J., CERVANTES, A. & 5 others (1989*a*). Quantitative determination of factors contributing to doxorubicin resistance in multidrug resistant cells. *J. Natl Cancer Inst.*, **81**, 1887.

SCHUURHUIS, G.J., PINEDO, H.M., CERVANTES, A. & 4 others (1989*b*). Mechanism of anthracycline resistance and its reversal in cells with high and low levels of multidrug resistance. *Proc. Am. Assoc. Cancer Res.*, **30**, 519 (abstract).

SLATER, L.M., SWEET, P., STUPECKY, M. & GUPTA, S. (1986). Cyclosporin A reverses vincristine and daunorubicin resistance in acute lymphatic leukemia in vitro. *J. Clin. Invest.*, **77**, 1405.

TSURUO, T., IIDA, H., KITATANI, Y., YOKOTA, K., TSUKAGOSHI, S. & SAKURAI, Y. (1984). Effects of quinidine and related compounds on cytotoxicity and cellular accumulation of vincristine and adriamycin in drug-resistant tumour cells. *Cancer Res.*, **44**, 4303.

TWENTYMAN, P.R. (1988). A possible role for cyclosporins in cancer chemotherapy. *Anticancer Res.*, **8**, 985.

WALKER, R.J., LAZZARO, V.A., DUGGIN, C.G., HARVATH, J.S. & TILLER, D.J. (1989). Cyclosporin A inhibits protein kinase C activity: a contributing mechanism in the development of nephrotoxicity. *Biochem. Biophys. Res. Commun.*, **160**, 409.

WILLINGHAM, M.C., CORNWELL, M.M., CARDARELLI, C.O., GOTTESMAN, M.M. & PASTAN, I. (1986). Single cell analysis of daunomycin uptake and efflux in multidrug-resistant and -sensitive KB cells: effects of verapamil and other drugs. *Cancer Res.*, **46**, 5941.

ZHAO, F.-K., CHUANG, L.F., ISRAEL, M. & CHUANG, R.Y. (1989). Cremophor EL, a widely used parenteral vehicle, is a potent inhibitor of protein kinase C. *Biochem. Biophys. Res. Commun.*, **159**, 1359.